# Overwintering Capacity of the Mediterranean Fruit Fly in the Dalmatia Region of Croatia

**DOI:** 10.3390/insects16111104

**Published:** 2025-10-29

**Authors:** Mario Bjeliš, Ivan Tavra, Frane Strikić, Luka Popović, Cleopatra A. Moraiti, Vasilis G. Rodovitis, Nikos T. Papadopoulos

**Affiliations:** 1 Department for Marine Studies, University of Split, Ruđera Boškovića 31, 21000 Split, Croatia; itavra@unist.hr (I.T.); fstrikic@unist.hr (F.S.); 2 Center for Plant Protection, Croatian Food Agency, Tisno bb, 20355 Opuzen, Croatia; l.popovic@hapih.hr; 3 Laboratory of Entomology and Agricultural Zoology, School of Agricultural Sciences, Department of Agriculture Crop Production and Rural Environment, University of Thessaly, Fytokou St., 38446 Volos, Magnesia, Greece; cmoraiti@gmail.com (C.A.M.); rodoviti@uth.gr (V.G.R.)

**Keywords:** hibernation, field survival, adult survival, pupae developmental duration, off season, longevity, lifespan, fecundity, overwintering refugia

## Abstract

The Mediterranean fruit fly, *Ceratitis capitata* (Wiedemann) (Diptera: Tephritidae) is considered one of the most important phytophagous pests worldwide. In Croatia, it severely affects the fresh fruit production in Dalmatia, and a national suppression action plan has been established to alleviate farmers’ burden. The aim of the current paper was to comprehensively study the overwintering dynamics of medfly in Dalmatia and investigate whether adults and pupae in addition to larvae can overwinter in different conditions, with a goal to support the establishment of a sound off-season control strategy against this pest. The off-season strategy, which was recently proposed in the frameworks of the EU Horizon 2020 funded project FF-IPM, focuses on the management of the low adult populations during winter and early spring period that has a detrimental effect on the development of the on-season summer and early autumn populations. Overwintering trials were conducted in Split (Dalmatia region, Croatia) using the local *C. capitata* population. Cages with different life stages (adults, pupae) or infested fruits were periodically transferred in three overwintering sites (open-field, semi-field, and urban conditions) from October to December (two to three establishment dates during the 2019–2020 and 2020–2021 season). Our results provide strong evidence that, in addition to larvae within fruit, both adult and pupae can survive in significant proportions in open-field and urban conditions. Overwintering site and establishment date were both significant predictors of the overwintering success of adults, and they affect pupae developmental duration, adult emergence rates from overwintering pupae, female fecundity, and generally the overwintering ability of the pupae. Our results suggest that the current national strategy for *C. capitata* suppression, which is based on the assumptions of larvae overwintering need to be adjusted by development and implementation of the off-season strategy.

## Simple Summary

To elucidate the field biology of the Mediterranean fruit fly (medfly), *Ceratitis capitata* (Wiedemann) (Diptera: Tephritidae), with the aim of developing robust pest management strategies, overwintering trials were conducted in Split, central Dalmatia, Croatia, between 2019 and 2021. Experiments confirmed the capacity of medfly to overwinter in the area and revealed that both adult and pupae can survive in open-field and urban conditions in central Dalmatia in addition to the overwintering of larvae in fruit. Overwintering site and the time individuals were exposed in field conditions (establishment date in autumn) determined the overwintering success of adults and affected pupae developmental duration, adult emergence rates from overwintering pupae, as well as the life history traits of yielded females. Urban sites can be recognized as additional hot spots for the overwintering of medfly in the area. Our results suggest that the current national strategy for *C. capitata* suppression, which is based on the assumptions of larvae overwintering, only needs to be adjusted by the development and implementation of the off-season strategies that were recently proposed in the framework of the EU Horizon 2020 funded project FF-IPM. The off-season strategy focusses on the management of the low adult populations during winter and early spring period that has a detrimental effect on the development of the on-season summer and early autumn populations of the pest.

## 1. Introduction

The Mediterranean fruit fly (medfly), *Ceratitis capitata* (Wiedemann) (Diptera: Tephritidae) is considered one of the most important invasive, phytophagous pests worldwide, affecting more than 300 different plant species [1,2], imposing a major threat for the fresh fruit production and trade. Originating in east, tropical Sub-Saharan Africa, medfly has dispersed and has become established on five different continents in temperate, subtropical, and tropical climates [3]. It was first reported in Africa in the Iberian Peninsula in 1842, Italy in 1863, and France in 1885, and subsequently spread along temperate areas of the Mediterranean countries [4,5,6,7]. Its great invasive characteristics, including high dispersal capacity, broad adaptability, and elevated reproductive potential, greatly enhance its ability to establish populations in new environments [5].

The distribution of the medfly has traditionally been considered restricted to below the 41st parallel north [8], and to habitats where temperatures regularly fall below 10 °C but not below 0 °C [9]. However, several publications demonstrated the capacity of medfly to overwinter as larvae within infested fruit in cold winter conditions and survive at temperatures below freezing [10,11]. Stable medfly populations have been reported in northern Greece [10,11], northern Italy [12,13,14,15,16,17], southern France [18], southern Germany [19], and Austria [20,21]. These data suggest that the species is capable of completing its life cycle and are probably established in areas far beyond its traditional northern range limit. Climate change [22,23], phenotypic plasticity [24,25,26,27,28,29], and the ability of the species to recolonize new areas with different agroecological conditions [30], through local transient populations [31], are expected to play a major role in expanding the existing distribution and performance of the species worldwide [25].

Climate change may alter the geographic limits of medfly, allowing its expansion into temperate areas of central Europe [32]. Physiological, morphological, and behavioral attributes enable medfly to survive and reproduce in a wide range of climatic conditions and habitats often well beyond the suggested thermal limits for development and adult performance [33,34]. Diamantidis et al. [35] determined that medfly populations originating from geographically isolated regions exhibit a high variation in their life history traits, such as longevity or fecundity, which may influence establishment and spread. Furthermore, Papadopoulos et al. [10,11] demonstrated that in northern Greece, medfly prolongs its larval development inside infested apples to cope with cold winter conditions. This overwintering capacity of introduced medfly populations can determine whether they can establish and grow in colder temperate areas [28,36]. Understanding the factors that allow *C. capitata* to adjust to such an enormous range of temperature variation may shed additional important light in elucidating the factors that promote its invasion success in cooler areas near to currently established areas [37,38,39,40].

*Ceratitis capitata* was first reported in Croatia in 1947 in Split, followed by several multiple detections along the Adriatic coast in the early 1950s [41]. Initial records on overwintering indicated adult emergence from the soil following pupation in early May [41]. Furthermore, this preliminary data revealed adult emergence from pupae during the November and December period followed by mortality of those adults by mid-March [42]. Nowadays, *C. capitata* is a pest of great economic importance in the Dalmatia region of Croatia, especially in the Neretva river valley, affecting production and exports of mandarins (*Citrus reticulata* Blanco). A national area-wide suppression program integrating the Sterile Insect Technique (SIT) method has been running on an operational level since 2013 [43]. The release of sterile flies is carried out from the middle of April until the end of November each year [44]. This strategy was based on the previously known data on *C. capitata* overwintering as larvae and the emergence of the first overwintering population in late April and May. Popović et al. [43] confirmed that developed larvae emerge from infested mandarins between October and the end of January, with peak emergence during November and December, serving as the overwintering stage in this area. However, the monitoring data of the population of adults and immature stages in the last few years has provided a new perspective on the situation with the *C. capitata* population in the Neretva valley and central Dalmatia during the winter period. Few single detections of *C. capitata* adults in April during 2014–2017 [45] and detection of fresh larva in *Fortunela kumquat* Tnub. fruits in mid-April 2016 indicate that adult populations are present in orchards during winter and infest the available host fruits [46].

As it is evident from the above, the information on overwintering dynamics of *C. capitata* in the Dalmatia area of Croatia is scarce and the existing information cannot explain fully the phenology patterns of adult captures. The current study deals with the overwintering capacity of the *C. capitata* in the area of Split and Solin located along the central Dalmatia coast of Croatia. Primary goals were to explore whether *C. capitata* adults and pupae in addition to larvae are capable of overwintering under different conditions (open-field, semi-field, and urban sites). We further seek to understand how knowledge on overwintering may help to plan a sound off season pest control strategy against this pest.

## 2. Materials and Methods

### 2.1. Insects Collection and Handling

Overwintering trials were conducted using adults and pupae from a local medfly population. Samples from the overwintering sites and the resident population in the region were used for molecular analyses over two consecutive years, revealing no significant differences between them within the same fruiting season [7]. Infested mandarins were collected from two locations: Dubrovačko primorje (Zaton: 42°42′07.0″ N 18°02′24.6″ E) and from Hvar island (Sućuraj: 43°07′32.7″ N 17°11′06.8″ E, Jelsa: 43°09′34.1″ N 16°41′27.4″ E, Starigrad: 43°10′52.4″ N 16°35′40.1″ E and Hvar: 43°10′16.7″ N 16°26′52.4″ E). Emerged pupae and adults were used for the overwintering trials. Wild, field-collected flies—rather than mass-reared individuals—were selected for the overwintering trials to avoid potential domestication effects associated with laboratory rearing techniques. Such effects, occurring during either the larval or adult stages, have been reported to influence overwintering capacity. The use of wild flies also allowed for a more accurate assessment of their natural ability to overwinter under near-natural conditions. Overwintering pupae and adults were periodically transferred in three overwintering sites in area of Split from October to December (three establishment dates during 2019–2020 and 2020–2021 season). During the 2019/2020 overwintering season, infested mandarins were collected for establishing the pupae overwintering experiments on 17 October 2019, 25 October 2019, and for the adult overwintering experiments on 19 November 2019, and on 12 October 2019, 2 November 2019, and 27 November 2019, respectively. During the 2020/2021 overwintering season, mandarins were collected and used for the pupae overwintering experiments on 9 October 2020, 13 November 2020, and for the adult overwintering experiments 21 December 2020, and on 15 October 2020, 18 November 2020, and 3 December 2020, respectively.

#### Fruits Handling and Larvae Collection for Adult and Pupae Overwintering Tests

Fruits were placed on a “collection table” fitted with a wire screen, allowing fully developed larvae to exit the fruit and fall into “collection boxes” positioned beneath the table. Tables with infested fruits were kept in the laboratory with controlled climatic conditions (24 °C, 65% relative humidity, and 12:12 L:D photoperiod) to accelerate development of larvae. Dates regarding fruit collection, larva collection and pupation were recorded for all establishment dates during two seasons. For adults overwintering experiments, pupae that were collected from infested fruits were kept on 25 °C and 50–55% relative humidity and exposed to the natural photoperiod conditions (L:D—11:13, 10:14 and 9:15 for three establishment dates in the 2019/2020 season and L:D—10:14; 10:14 and 9:15 for the establishment dates during 2020/2021 season).

The age of pupae corresponding to three establishment dates per season, ranged from 2 to 4 days post-pupation across different seasons and dates. Adults exposed to various overwintering conditions were 2–3 days old in the 2019/2020 season and 3–5 days old in the 2020/2021 season.

### 2.2. Overwintering Sites

**Figure 1:**
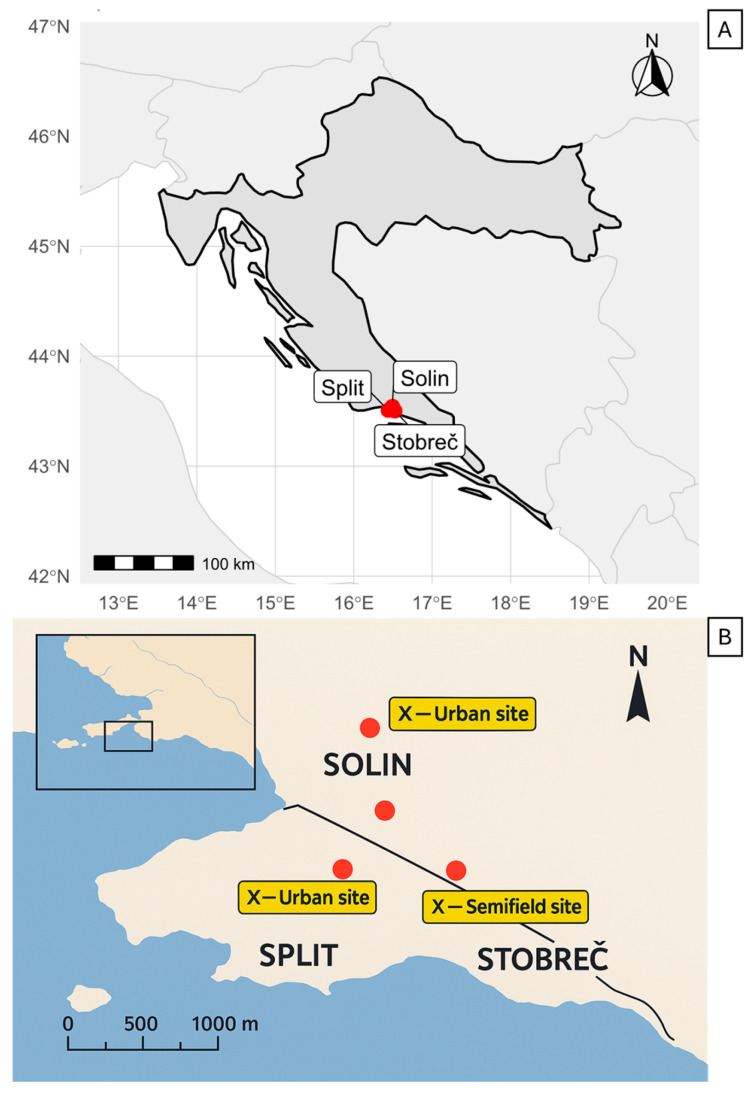
Map showing (**A**) the locations of the experimental sites used for the overwintering experiments and (**B**) the location of the urban and semi-field sites. Map created in RStudio (R version 4.5.1).

Overwintering trials were conducted in the cities of Split and Solin (Dalmatia region, Split—Dalmatia County of Croatia) during two seasons (2019–2020 and 2020–2021) (Figure 1).

#### 2.2.1. Outdoor Conditions (Open-Field Conditions)

The experimental site [0.23 ha mixed orchard of several types of fruit plants (fig—*Ficus carica* L., olives—*Olea europaea* L., walnuts—*Juglans regia* L., cherries—*Prunus avium* L. and almonds—*Prunus dulcis* L.)] is located in the town of Solin (43°31′57.7″ N 16°29′57.7″ E and 30 m above sea level). This site is a typical backyard in which many plant species of different ages are grown in an extensive way and without any planting structure. The cultivation was very extensive, consisting of mulching of the weeds with a mower once or twice a year. On this surface, no pesticides or fertilizers were applied. As a position for the placement of cages, a site was selected along the edge of the plot between one cypress—*Cupressus sempervirens* L. tree and two olive trees in a place sheltered from winds and direct sun light. Sand bottles and adult cages were placed directly on the ground, while individual cages with one pair of adults were placed in a shallow lattice basket due to wind sensitivity. Cages were protected from rain by a transparent polyethylene cover suspended horizontally 1 m above the cage top. A data logger for collecting climate data (temperature, relative humidity) was attached to the wooden girder.

#### 2.2.2. Storehouse in Field (Semi-Field Conditions)

This site is located on the eastern periphery of the town of Split in the village Stobreč (43°30′28.8″ N 16°31′33.4″ E and 18 m above sea level). It was a ground floor building of simple construction with a total area of 50 m^2^ without heating source. The warehouse has a large tin garage door on one side and two windows 20 × 60 cm on the other side for ventilation. This warehouse was used for storage of various types of tools, equipment, and agricultural devices and various types of containers for handling agricultural products. Ventilation of this room is continuous and access to light is possible through the windows and varies from 50 to 85 Lux. For the needs of the research storage shelves were used to place individual cages, sand bottles, or adult cages. For the purpose of inspecting the cage, 10 V halogen lamps were installed, which were in operation only during inspection. Temperature and relative humidity in the room was recorded with a data logger placed on the shelves hosting the different *C. capitata* developmental stages.

#### 2.2.3. Basement of a Building (Urban Conditions)

This site is located in the urban area in the town of Solin, in a basement of the five floors building (43°32′02.3″ N 16°29′52.1″ E and 16 m above sea level) without heating. A room of 20 m^2^ in size without heating and with two small openings of 25 × 50 cm in the upper part, which are used for ventilation, was used. Access to daylight in the room is very limited, and always below 20 Lux. This is a typical room in the Dalmatia region of Croatia that is used to store different types of agricultural products throughout the year, such as potatoes, fruits and vegetables, processed products such as olive oil, wine, etc., and further different types of packaging, bottles, clothing, and footwear they do not use. For the needs of the experimental trials, a wooden support was constructed in the room, which was attached to the wall, and aluminum portable shelves on which individual cages, sand bottles, or cages for adults were placed. To facilitate cage inspection and data recording, 5 or 10 V halogen lamps were installed, which were switched on only during inspections. Temperature and relative humidity in the room was recorded with a data logger placed on the shelves hosting the different *C. capitata* developmental stages.

### 2.3. Experimental Design

#### 2.3.1. Adults Overwintering Trials

For adult overwintering experiments, pupae collected from infested fruits were placed in 30 × 30 × 30 cm Plexiglas cages and maintained in laboratory until adult emergence. For each treatment and establishment date, 50 males and 50 females (one day-old) were placed into 10 small plastic cages (25 × 15 × 15 cm) with wire screen top and both sides (5 males and 5 females/cage) and transferred to the three overwintering sites at 30 October 2019 (1st date), 19 November 2019 (2nd date) and 18 December 2019 (3rd date) (10 cages/site/date) during season 2019–2020 and 6 November 2020 (1st date) and 14 December 2020 (2nd date) during season 2020–2021 (Table 1). Hence, 10 cages were established in each overwintering site. Insects were provided with water and adult food (a 1:4:5 mixture of yeast hydrolysate, sugar and water). Cages were transferred to different overwintering sites and inspected at least twice a week to count and remove dead adults.

#### 2.3.2. Pupae Overwintering Trials

A total of between 20 and 40 pupae (2–5 days old) were introduced into a cylindrical plastic bottle (1 L; 30 cm high, 10 cm diameter) bearing a side ventilation window (5 cm diameter, covered with tulle) opened in the protruding part of the bottle. Pupae were placed on the surface of a 5 cm layer of soil and covered by 10 cm of soil. In order to prevent predation, the bottles were capped. Bottles with pupae were transferred into the three overwintering sites on 28 October 2019 (1st date), 6 November 2019 (2nd date), and 5 December 2019 (3rd date) (10 bottles/site/date) during the experimental season 2019–2020 and 16 October 2020 (1st date), 21 November 2020 (2nd date), and 30 December 2020 (3rd date) during 2020–2021 (Table 2). The bottles were inspected to record emerging adults every two days. Emerged adults were sexed, counted, and then removed. Upon emergence, adults were sexed, and the emergence date was recorded. Adults ordered by day of emergence (one male and one female per cage) were placed in individual cylindrical plastic cups (0.5 L; 12 cm high, 8 cm diameter) with a 5 cm diameter opening covered with tulle and maintained at the same site. Females were allowed to oviposit on 5 cm-diameter hollow, plastic hemispheres of red color (domes) that were artificially punctured with 40–50 evenly distributed holes on their surface. Eggs were deposited on the inner surface of the dome. Each dome was fitted in a 5 cm-diameter hole made on the cover of a 5.5 cm-diameter plastic Petri dish. Flies had full access to standard adult diet drops consisting of a mixture of yeast hydrolysate, sugar, and water (YS) at a 4:1:5 ratio, respectively. Water was placed in the base of the Petri dish in order to maintain humidity levels beneath the dome adequate enough for female oviposition. A plastic cup containing 0.5 mL of orange juice was also placed in the base of the Petri dish to stimulate oviposition. Mortality and female fecundity as the number of eggs/female (for 2019–2020 season only) were daily recorded. Longevity and reproduction were recorded following Diamantidis et al. [35].

### 2.4. Statistics

The Cox proportional hazard model was used to assess (a) the effects of overwintering site, establishment date and sex on survival patterns of overwintering adults, and pupae-to-adult developmental time for adults emerged from overwintering pupae, and (b) the effects of overwintering site and establishment date on female lifespan for females that emerged from overwintering pupae. Significant factors were entered into a multifactorial Cox regression model using a forward stepwise procedure for model selection. Nonsignificant factors (Wald’s test) were excluded from the final model. Binary logistic regression analysis was used to assess the effects of overwintering sites, and establishment dates on adult emergence rates of overwintering pupae. Statistical analysis was performed with SPSS v.26.

## 3. Results

### 3.1. Climatic Profile of the Overwintering Sites

**Figure 2:**
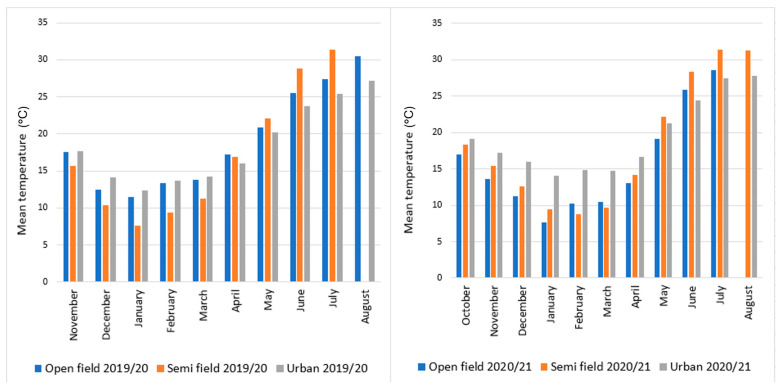
Mean monthly temperature for each overwintering site (open-field, semi-field, and urban conditions) from November 2019 to August 2020 and from October 2020 to August 2021. Overwintering trials were initiated between October and December in both years, and flies survived until spring.

**Figure 3:**
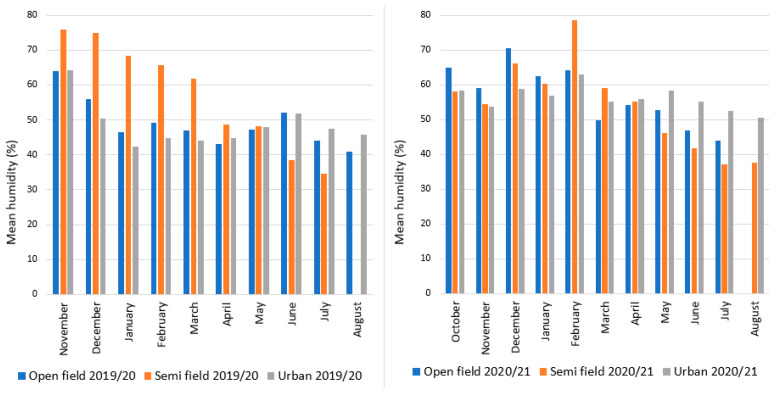
Mean monthly humidity for each overwintering site (open-field, semi-field, and urban conditions) from November 2019 to August 2020 and from October 2020 to August 2021. Overwintering trials were initiated between October and December in both years, and flies survived until spring.

Detailed climate data sets for all overwintering sites in Croatia for both seasons are given in Figure 2 (temperature) and Figure 3 (humidity). Overall, the temperature was warmer in the urban site during winter compared to both open-field and semi-field site, while differences between the last two depend on the year. Humidity during winter in all three sites ranged from 40 to almost 80% during winter in all three sites and it was higher during the second experimental period. In the first experimental period, humidity was higher in the semi-field site compared to both open-field and urban sites. However, differences among sites were negligible in the second experimental season.

### 3.2. Adults Overwintering

The average life span of males and females in three overwintering sites for each establishment date is given in Table 3. In 2019, females outlived males in all three overwintering sites and dates, except for those transferred to semi-field conditions on 19 November 2019. Males outlived females during 2020 season. Patterns of longevity in open-field and urban conditions, although dependent on the establishment date, were rather unpredictable, apparently reflecting variability in weather conditions. A clear pattern of shorter longevity for both males and females at later exposure dates were found in the semi-field conditions.

Cox regression analysis revealed that overwintering site, establishment date, and sex were significant predictors of adult survival in Split throughout the winter periods of 2019–2020 and 2020–2021 (Table 4).

**Figure 4:**
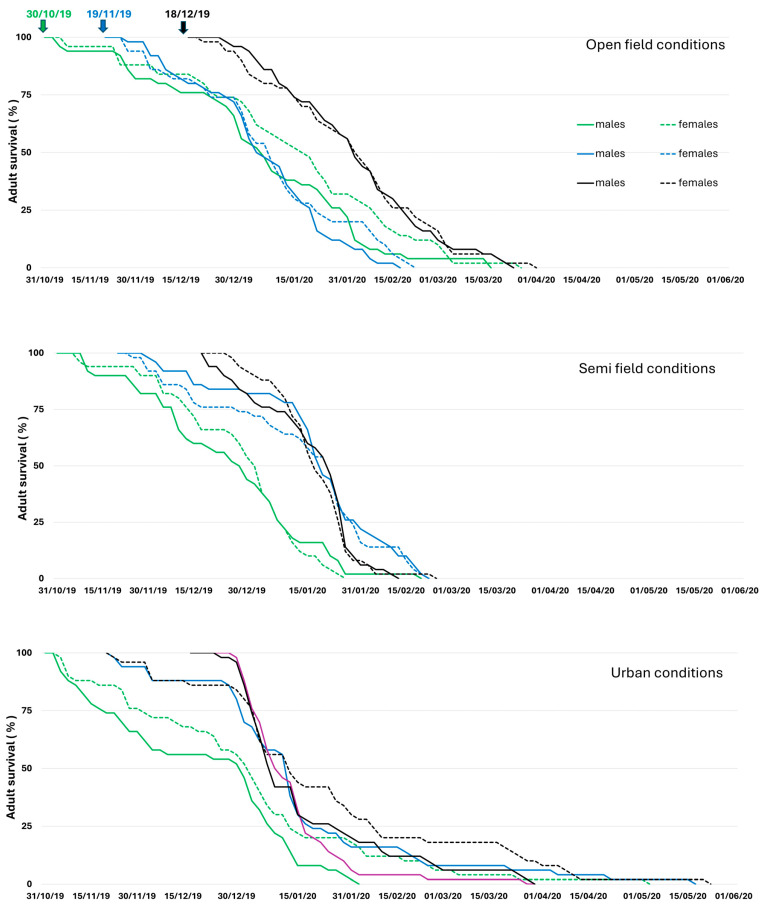
Survival patterns of *Ceratitis capitata* males (solid line) and females (broken line) that were transferred in three overwintering sites on 30 October 2019, 19 November 2019, and 18 December 2019 during the winter season 2019–2020. Males and females followed similar mortality patterns in most of the cases and the establishment date had more dramatic effects in urban conditions compared to semi-field and open-field conditions. More individuals managed to survive up to spring in urban conditions followed by open-field conditions.

**Figure 5:**
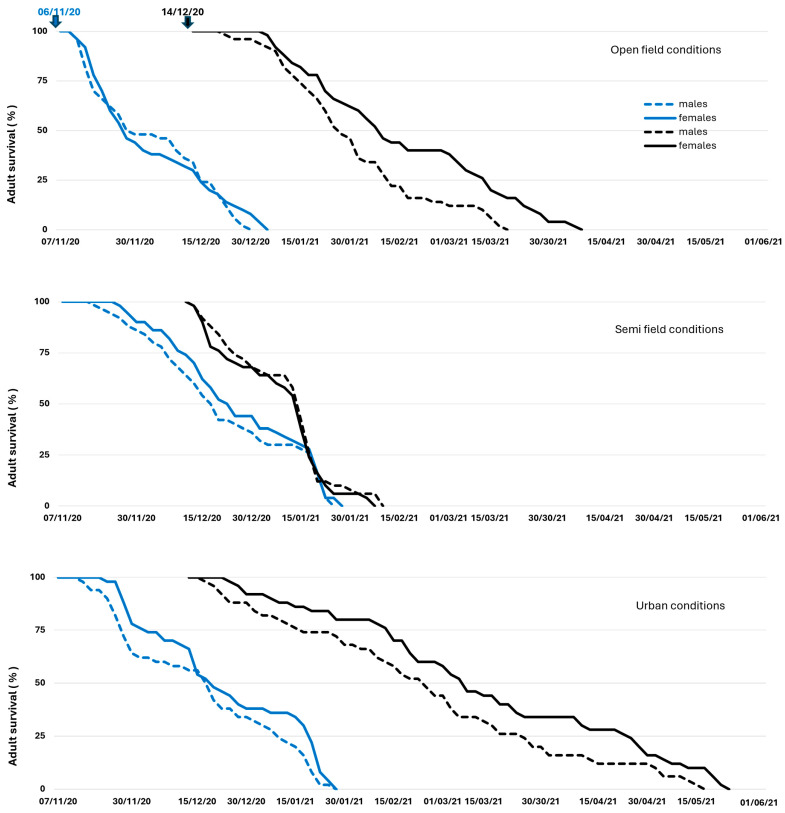
Survival patterns of *Ceratitis capitata* males (solid line) and females (broken line) that were transferred in three overwintering sites on 6 November 2020 and 14 December 2020 during the winter season 2020–2021. Males and females followed similar mortality patterns in most of the cases and the establishment date had more dramatic effects in semi-field conditions compared to urban and open-field conditions. More individuals managed to survive up to spring in urban conditions followed by open-field conditions.

Adult mortality rates were very low until mid- December for flies remaining in open-field and semi-field conditions. Mortality rates were higher for flies kept in urban conditions for all three establishment dates during the 2019/20 season and for the first establishment date (December) during the 2020/2021 season (Figure 4 and Figure 5).

During January–February of the 2019/2020 season, there was a sharp increase in mortality rates for adults that remained under semi-field and urban conditions for all three establishment dates. In contrast, mortality rates were increasing progressively for flies in open-field. During the 2020/2021 season, the mortality rates were very high for the first establishment date on all three overwintering sites and adults did not manage to survive until the end of January. On the contrary, adults from the second establishment date (December) in open-field and urban conditions were capable of surviving until spring (April–May). All flies in semi-field conditions died up to the end of February during both seasons, implying that flies were not capable of surviving until spring. This can be attributed to a general lack of sunlight benefits and unfavorable temperatures that were below the *C. capitata* developmental threshold of approximately 10 °C (January and February of 2020 and January–March of 2021). In contrast to semi-field conditions, flies kept under urban conditions survived up to the spring period (from March to May) in all three establishment dates during the 2019/2020 season, and for one (14 December 2020) of the two establishment dates during the 2020/21 season. In open-field conditions, flies managed to survive up to the end of March for the establishment dates of the October and December 2019/20 season and through April for the establishment dates of December 2020/21.

In addition to mortality rate, the cumulative survival patterns of adults as a function of age is given in Figures S1 and S2 for the two seasons, respectively. Both males and females lived longest in urban conditions in 2019 and 2020. Females in urban conditions lived up to 181 and 188 days in October and November 2019 establishment dates and up to 157 days in December 2020 establishment date. Males in urban condition lived up to 180 days in November 2019 and 143 days in December 2020 establishment dates, respectively. This can be attributed to temperatures that were favorable for adults and ranged between 13 and 17 °C and 14–16 °C for the December–February period for both seasons in urban conditions.

The longest life span for males (138 days) and females (142 days) in open-field conditions was recorded on the October 2019 establishment date. The longevity of females ranged between 100 and 110 days on the November and December 2019 and December 2020 establishment dates. The male life span ranged between 95 and 100 days in November and December 2019 and only 55–80 days during the 2020 establishment dates. The longest life span of females in the semi-field conditions ranged from 75 to 115 days on the 2019 establishment dates and between 80 and 110 days on the 2020 establishment dates. The male life span in semi-field conditions ranged from 61 to 105 days on the 2019 establishment dates and 78–82 days on the 2020 establishment dates. The short longevity of adults in semi-field conditions could be explained by the impact of non-favorable temperatures that were below 10 °C during January and February 2020, which is the same as during the January–March period of 2021.

Adults overwintering in different conditions was demonstrated by the proportion of individuals that succeeded in surviving winter and entered the spring period. In open-field conditions, 4–6% of males and 2–6% of females succeed in surviving the winter period for the October and December 2019 establishment dates (Figure 4) and 10% of males and 26% of females on the December 2020 establishment date (Figure 5). In urban conditions, 4–18% of females and 2–8% of males survived winter in all three establishment dates in 2019 (Figure 4), and 42% of females and 33% of males on the December 2020 establishment date, respectively (Figure 5). On the contrary, the adults in semi-field conditions died before the end of February for all establishment dates for both years (Figure 4 and Figure 5).

### 3.3. Pupae Overwintering

**Figure 6:**
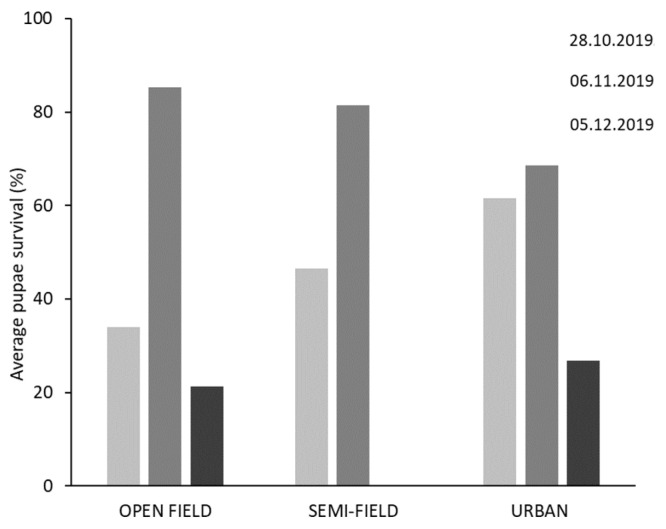
Effect of establishment date and area of exposure on pupae winter survival (indicated by adult emergence) on season 2019/20. Pupae in open-field conditions expressed similar average survival in most of the exposure dates with semi-field while these in urban areas were, in general, higher regardless of the establishment date.

**Figure 7:**
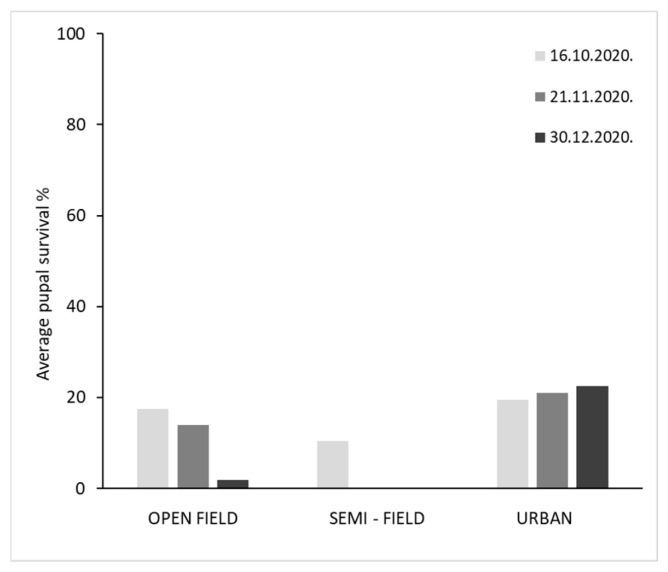
Effect of establishment date and area of exposure on pupae winter survival (indicated by adult emergence) on season 2020/21. Pupae in open-field conditions expressed similar average survival in most of the exposure dates with semi-field while these in urban areas were, in general, higher regardless of the establishment date.

Adult emergence rates from overwintering pupae in the three overwintering sites for each establishment date are given in Figure 6 and Figure 7. During the 2019/20 season, high emergence rates were recorded for pupae transferred to the different overwintering sites early in November. In contrast, low emergence rates were recorded for pupae transferred to the different overwintering sites early in December. No adults emerged from semi-field conditions for the December establishment date. During the 2020/21 season, emergence rates in all three sites were lower than in the previous year. High emergence rates were recorded for pupae transferred to the different overwintering conditions in October, and generally for those placed in urban site across all three establishment dates. No adults emerged from semi-field conditions for the November and December establishment dates.

In general, the second date of establishment (November) in 2019–2020 yielded the best overwintering results across all three sites. During the first two experimental dates of the trial in 2019–2020, survival was >60% under urban conditions. In the open-field, the survival rate was >80%. Urban conditions proved to be the most favorable overwintering site, with a high percentage of surviving individuals across all three establishment dates in both seasons. In contrast, pupal survival on the third establishment date (December) was the lowest at all three sites, approximately 20% in open-field and urban conditions, while pupae did not develop in the semi-field conditions.

In the 2020–2021 season, the proportion of overwintering pupae was generally lower than in the 2019–2020 season. For example, pupae did not survive under semi-field conditions on the second and third establishment date of the experiment, confirming that temperatures below 10 °C that prevailed after the second and third establishment dates were unfavorable for pupae development in semi-field sites. The same conditions also affected the pupae development for the third establishment data in open-field conditions. In contrast, pupae survived under field conditions in the first two establishment dates and in urban conditions. Urban conditions give the highest percentage of overwintering. Binary logistic regression analysis revealed that overwintering site and establishment date had a significant effect on pupae survival confirmed by adult emergence (Table 5).

Cox regression analysis showed that overwintering site, establishment date, and interactions were significant predictors of pupae developmental duration (Table 6).

**Figure 8:**
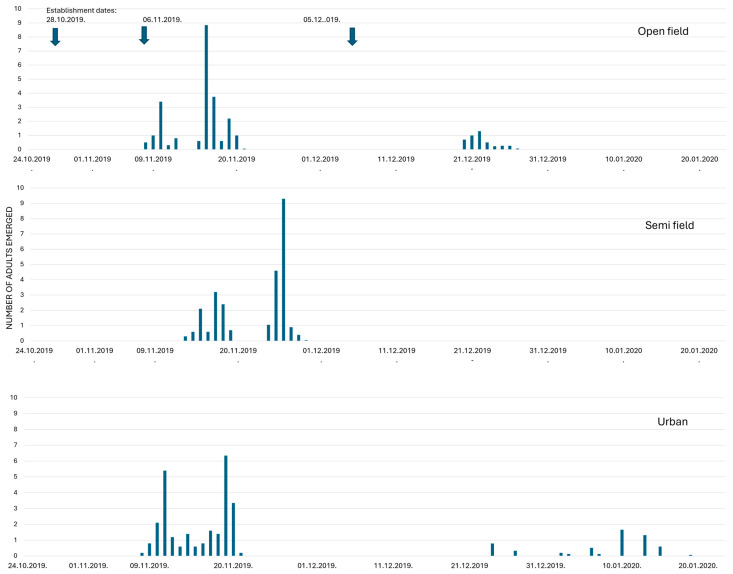
Temporal patterns of adult emergence from overwintering pupae that were placed on three sites (open-field, semi-field, and urban conditions) at three establishment dates in the 2019/2020 season. The number of emerged adults was higher when pupae were exposed in autumn compared to exposure during the winter.

**Figure 9:**
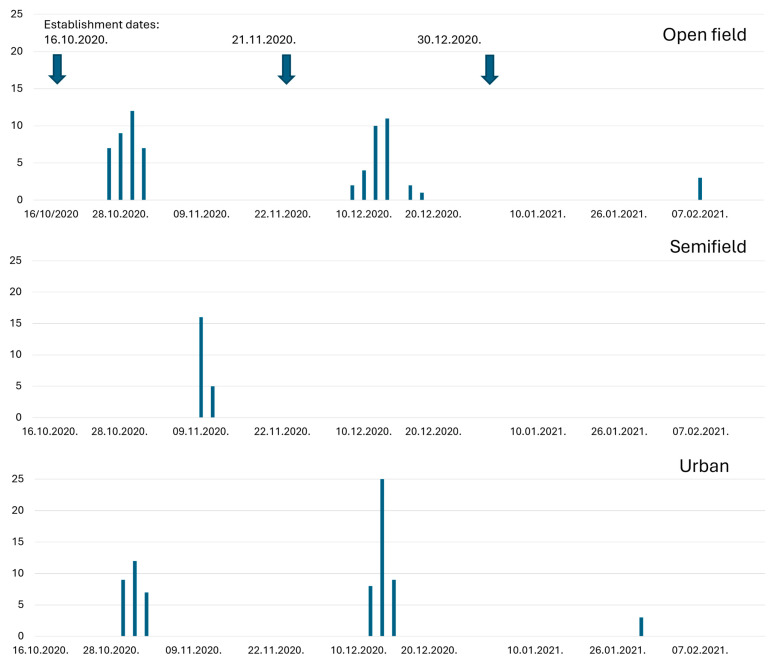
Temporal patterns of adult emergence from overwintering pupae that placed on three sites (open-field, semi-field, and urban conditions) at three establishment dates in the 2020/2021 season. The number of emerged adults was higher when pupae were exposed in autumn compared to exposure during the winter.

The temporal pattern of emergence of *C. capitata* adults from overwintering pupae for three establishment dates on three overwintering sites is presented in Figure 8 and Figure 9. In 2019–2020, favorable conditions prevailed at all three overwintering sites during the periods of the first two establishment dates. Patterns of adult emergence in open-field and urban conditions were similar. In semi-field conditions the developmental duration of pupae was slightly prolonged for the first two establishment dates. In contrast, developmental duration of pupae at the third establishment date was prolonged in the open-field and urban conditions. No pupae survived in semi-field conditions, apparently due to low temperatures (<10 °C from December to March (Figure 8).

Similar patterns in adult emergence at the different overwintering sites were confirmed during 2020–2021 (Figure 9). Pupae did not complete development and hence did not overwinter under semi-field conditions during the second and third establishment dates. In summary, both establishment date and overwintering site affected pupae developmental duration, adult emergence rates and hence the overwintering ability of the pupae.

#### Adult Demographic Traits

Females lived longer under open-field conditions and urban conditions, regardless of the establishment date than females in semi-field conditions (Table 7). No pupae yielded adults in the third establishment date in semi-field conditions.

**Figure 10:**
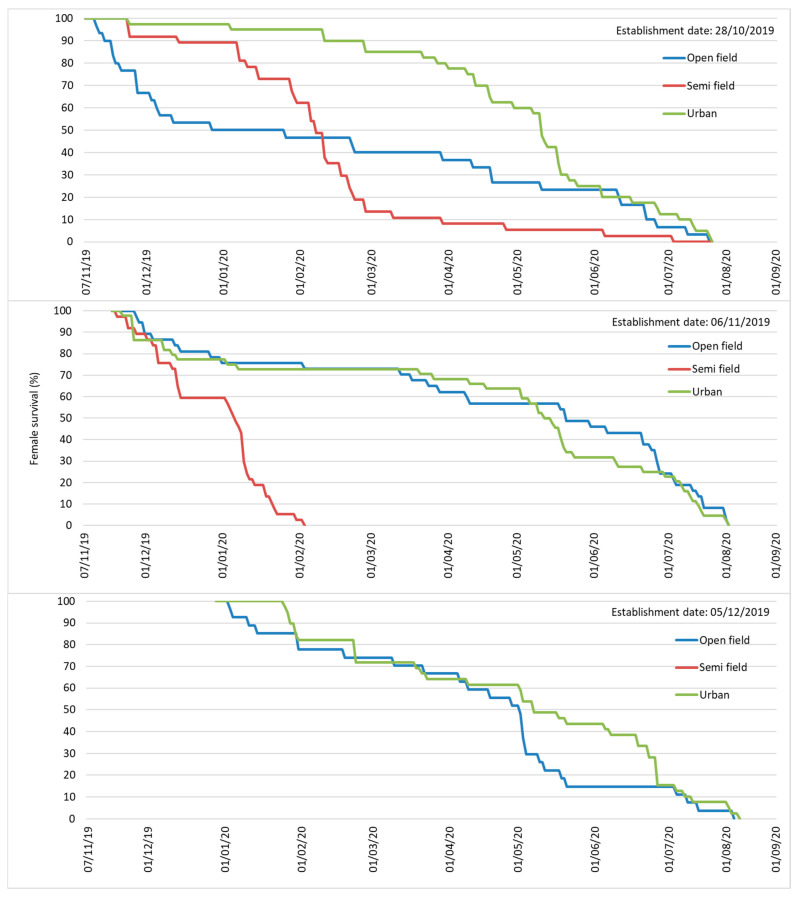
Age-specific survival patterns of females emerged from overwintering pupae in the three overwintering sites for each establishment date (first: 28 October 2019, second: 6 November 2019, third: 5 December 2019). Females remained in the same overwintering sites as pupae from which were obtained. Females survived up to the middle of July from all sites, while those developed as pupae in open-field and urban conditions survived in higher numbers.

Cox regression analysis revealed that overwintering site was a significant predictor of female lifespan (*χ*^2^ = 51.320, df = 2, *p* < 0.001), whereas the establishment date was not (*χ*^2^ = 3.511, df = 2, *p* = 0.173). Age specific survival patterns of females emerged from pupae in open-field conditions were exceptionally high for 4 to 5 months especially for those reaching adulthood during November (Figure 10). Approximately 40 to ≈60% of the females survived until April 2020 and 15 to ≈45% until June 2020, which represents a significant potential of pupae overwintering during all three establishment dates. A substantial proportion of females in open-field conditions lived longer than 8 months (240–250 days) until the end of July and even until the beginning of August 2020.

Survival patterns of females that emerged from pupae in urban conditions showed a similar trend to those in open-field conditions, with 65 to ≈ 80% surviving until April 2020, and 25 to ≈40% surviving until June 2020, reaching the maximum lifespan of 240–250 days by the first decade of October.

In contrast to open-field and urban conditions, there was sharp decrease in survival of females in the second establishment date leading to no survivors by February 2020. On the third establishment date, no adults emerged from pupae. Females that emerged from pupae placed in semi-field conditions on the first establishment date in October lived more than 240 days.

Average female fecundity was extremely low in all overwintering sites (Table 7). The highest fecundity was observed for the females from the third establishment date in open-field conditions. The proportion of ovipositing females differed between sites and establishment dates. In open-field conditions, 13.33 (first establishment date), 18.92 (second establishment date), and 4.54% (third establishment date) of females laid eggs. In semi-field conditions, no oviposition was observed, while in urban conditions 11.42% (first establishment date), 5.12% (second establishment date) and 6.56% (third establishment date) of females produced eggs.

**Figure 11:**
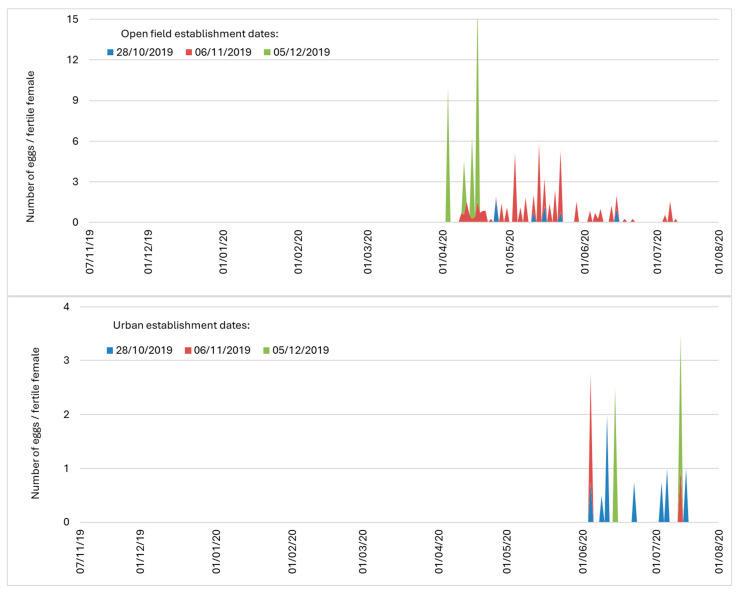
Egg laying patterns of females emerged from overwintering pupae in the three overwintering sites for each establishment date (first: 28 October 2019, second: 6 November 2019, third: 5 December 2019). Females remained in the same overwintering sites as pupae from which were obtained.

The egg laying period in the open-field conditions extended over three months, from the beginning of April 2020 until the end of July 2020 (Figure 11). Females that emerged from pupae on the first establishment date laid eggs from mid-April up to late May 2020. Females that emerged from pupae on the second establishment date laid eggs from mid-April till the end of July 2020. Females emerging from pupae of the third establishment date laid eggs only during first 10 days of April 2020, although they laid the highest number of eggs/fertile female. The egg laying period in urban conditions lasted even two months later than in open-field conditions and was much shorter in duration (beginning of June until mid-July 2020) with similar trends of egg laying patterns among the establishment dates.

## 4. Discussion

Our findings show that *C. capitata* successfully overwinters mainly as adult and pupae in both open-field and urban environments in Central Dalmatia, Croatia. A significant proportion of adults survived winter in these environments, especially when established in November, while those in semi-field conditions died by late February in both years. Adult survival lasted up to 4 months in open-field and 6 months in urban areas. Urban sites had the highest overwintering success, particularly for adults, whereas pupae established in December had the lowest survival. Adult emergence patterns suggest that temperatures were favorable during the first two establishment dates across all sites, though emergence was delayed in semi-field and urban conditions when pupae were established in December. No emergence occurred under semi-field conditions during this period, likely due to temperatures below the 10 °C developmental threshold. Females lived longer in open-field and urban environments and showed high spring–summer survival. Oviposition in the open-field extended from early April to mid-July, with varying peaks, while in urban sites it spanned early June to mid-July with a more uniform trend. Results suggest the clear overwintering capacity of *C. capitata* in northern temperate parts of Europe such as Croatia.

Adults of *C. capitata* have not been considered in the past as the common overwintering life stage in the temperate regions. The failure of the fly to overwinter as an adult has been reported in Thessaloniki, Greece (40° N) [10], in Girona, Spain (42° N) [47], and in Vienna, Austria (48° N) [48]. In the southern part of the Euro-Mediterranean region, studies showed that adults persist only until January in Chios, Greece (38° N) [49], and until December in Thessaloniki, Greece [11], while successful overwintering occurs only in mild-winter regions like Crete, Greece (35° N) [50]. The latter also found that adding a live branch improved survival by offering protection from rain and wind. In nature, flies shelter under citrus canopies, where temperatures are 1–2 °C higher than ambient temperatures [51]. In our study, adult cages were placed in a roofed, double-netted structure to shield them from wind and rain. However, the overwintering capacity and the specific overwintering stage of each *C. capitata* population are influenced by their geographic origin and underlying genetic variation, both of which are closely associated with the microclimatic conditions of their native regions [35,52].

Except for the protection, acclimation period to low temperatures in the adult stage found to reduce the critical thermal minimum (CTmin) and the time to recover from chill comma, while it extended the survival of the pest. In our study, most adults that obtained from overwintering larvae in fruits expressed very short lifespan in open-field conditions, while the lifespan of adults in urban conditions last approximately eight months highlighting the importance of urban area as overwintering hot-spots where the temperature is not very low and provides the time for the flies to acclimate and express their tolerance. The adult stage of *C. capitata* was found to be the most susceptible to low temperatures, as critical thermal minimum ranges from 5.4 to 6.6 °C [38,53,54]. On the other hand, thermal tolerance is affected by the acclimation period and temperature; for example, 5 days at 10 °C lowered the Lower Lethal Temperature (LLT) and improved survival according to Papadogiorgou et al. [55]. In our study the mean temperature was almost 10 °C most of the period, which means that the flies were able to acclimatize, while it was above the CTmin reported thresholds, which suggest that adults were able to move, feed, and search for shelters especially during the warm days, providing themselves the chance to survive during the winter.

Overwintering of *C. capitata* in the Dalmatia region does not depend exclusively on the capacity of larvae to survive the low temperature extremes during the coldest winter months as has been demonstrated for Northern Greece [10,11]. Our research strongly confirms the indication that adult population could be present in orchards during winter and oviposit in the available host fruits [46], after single detections of *C. capitata* adults in April during 2014–2017 period [45] and detection of fresh larva of *C. capitata* in *Fortunela qumquat* fruits in mid-April 2016 [46]. A significant proportion of the *C. capitata* population that enters the winter season as adults or adults that were developed from overwintering pupae is capable of surviving unfavorable temperature conditions and remaining alive through the spring and summer period in open-field and urban conditions. The acclimation period on the adult stage explained above in combination with the developmental acclimation in the immature stages plays a key role in the cold tolerance and overwintering capacity of insects and especially fruitflies. Developmental duration is mainly determined by the accumulation of degree-days although it is also strongly influenced by sub-threshold thermal conditions [39] and studies cited in. Exposure to temperatures below the developmental threshold can prolong the duration of immature stages and impose physiological costs that ultimately affect both survival and reproductive potential of adults. Such interactions between thermal accumulation and stress physiology provide a more realistic framework for interpreting overwintering success. Warm developmental temperatures often accelerate development but may lead to the production of individuals poorly equipped to tolerate cold stress, reducing their overwintering success [56,57]. In contrast, low developmental temperatures typically prolong development and have been shown to induce phenotypes with enhanced cold tolerance [56,58,59,60,61]. These plastic responses are particularly important for understanding the invasive potential and seasonal dynamics of tephritid fruitflies in temperate regions [62].

Recent research has elucidated temperature as the main factor driving the establishment of *C. capitata* also in areas farther north than the 41st parallel in Europe [25], and the city of Split (43rd parallel) in Dalmatia region of Croatia is a the confirmed example of this. Further consideration predicts a shift in the dispersal of *C. capitata* up to the 46th parallel north in a continental climate in the north of Italy and France [25]. Surveys conducted in the Dalmatia region of Croatia during 2020 and 2021 showed that *C. capitata* has spread and invaded large parts of the sub-Mediterranean inland areas of Split–Dalmatia and Šibenik–Knin counties, reaching up to the 44th parallel [63]. However, further overwintering studies are still needed to confirm the establishment of the pest.

In the light of our findings, new approaches exploring whether off-season strategies and suppression methods to control the low adult populations of the overwintering generation should be considered [64]. Such change can result in a suppression of population that is very low during winter and early spring. Current suppression methods of *C. capitata* in area wide control programs is based on a variety of tools ranging from pesticides, mass trapping, the Sterile Insect Technique (SIT) and the use of biological control, the latter involving predators, parasitoids, and entomopathogens [43,64,65,66,67]. Based on the results of this research, optimal releasing time of the sterile males seems to be earlier than late April since adult population is present through winter and spring, as already suggested by Bjeliš et al. [45]. Low doses of commercial EPN can be used for pest suppression off-season and early season in order to contain *C. capitata* populations before they grow significantly as the season progresses [68,69]. Several studies confirm the effectiveness of the mass trapping method to target low *C. capitata* populations early in the season and under low temperature conditions. Furthermore, female selectivity of mass trapping devices is also equally important for success of mass trapping control since maximal number of females could be removed from field populations before attacking fruits [64,68,69,70,71,72].

## 5. Conclusions

Experiments confirm that a significant proportion of *C. capitata* population overwinter as adults and pupae in the open-field and urban conditions in central Dalmatia in addition to overwintering of larvae in fruit, as previously confirmed. Overwintering site and establishment date are both significant for overwintering success of adults and significantly affect pupae developmental duration, adult emergence rates from overwintering pupae, female fecundity, and generally the overwintering ability of the pupae. The lifespan of adults confirms that a significant part of the population is present in the open-field and in urban conditions during the entire period from the beginning of winter through spring, while they are able to mate and lay eggs in the fruits of available host plants. Urban sites can be recognized as additional hot-spots. Findings from this research suggest that current national suppression strategy for *C. capitata* management, which assumes overwintering occurs only in the larval stage, needs to be revised. The strategy should incorporate the development and implementation of the off-season pest management strategies and suppression methods aimed at targeting low adult populations during winter and early spring period.

## Figures and Tables

**Table 1:** Experimental design and setup for the adult overwintering trials.

Adult Overwintering	Number of Replications	
Site/Season	2019/2020	2020/2021
Open-field	10 × 3 establishment dates: 30 October 2019, 18 November 2019, 18 December 2019	10 × 2 establishment dates 6 November 2020, 14 December 2020
Semi-field	10 × 3 establishment dates: 30 October 2019, 18 November 2019, 18 December 2019	10 × 2 establishment dates 6 November 2020, 14 December 2020
Urban	10 × 3 establishment dates: 30 October 2019, 18 November 2019, 18 December 2019	10 × 2 establishment dates 6 November 2020, 14 December 2020

**Table 2:** Experimental setup details for pupae overwintering experiments.

Establishment Dates			Replications (Pupae/Replication)	
Site	Season 2019/20	Season 2020/21	Season 2019/2020	Season 2020/2021
Open-field	28 October 2019 6 November 2019 5 December 2019	16 October 2020 21 November 2020 30 December 2020	10 (20) 10 (40) 10 (30)	10 (20) 10 (20) 9 (20)
Semi-field	28 October 2019 6 November 2019 5 December 2019	16 October 2020 21 November 2020 30 December 2020	10 (20) 10 (40) 10 (30)	10 (20) 10 (20) 10 (20)
Urban	28 October 2019 6 November 2019 5 December 2019	16 October 2020 21 November 2020 30 December 2020	10 (20) 10 (40) 10 (30)	10 (20) 10 (20) 8 (20)

**Table 3:** Average lifespan of overwintering *Ceratitis capitata* adults in three sites located in Split (Dalmatia region, Croatia) during the winter season 2019–2020 and the winter season 2020–2021.

Establishment Date	Average Life Span (Days ± SE)					
	Open-Field Conditions		Semi-Field Conditions		Urban Conditions	
	Males	Females	Males	Females	Males	Females
30 October 2019	68.7 ± 4.3	77.8 ± 4.5	55.4 ± 3.3	58.1 ± 2.7	48.1 ± 3.8	63.1 ± 5.2
19 November 2019	51.2 ± 2.7	53.1 ± 3.3	63.3 ± 3.0	56.5 ± 3.6	60.6 ± 4.8	69.1 ± 5.6
18 December 2019	46.2 ± 3.0	47.0 ± 3.4	30.2 ± 2.1	31.2 ± 1.6	27.1 ± 2.1	31.6 ± 3.3
6 November 2020	27.1 ± 2.3	26.9 ± 2.4	49.2 ± 2.6	47.9 ± 3.0	46.0 ± 3.1	43.5 ± 3.4
14 December 2020	64.4 ± 3.9	50.3 ± 3.2	26.4 ± 2.1	27.3 ± 2.0	89.8 ± 5.9	71.0 ± 5.7

**Table 4:** Explanatory variables of the Cox proportional hazard model on the survival of overwintering adults. “*B*” is the model coefficient and “exp (B)” shows the relative risk of adult survival at any given time. Males transferred to the urban site on 18 December 2019 (season 2019/2020, top) and on 14 December 2020 (season 2020/2021, below) from the baseline.

Source of Variance	*B* ± SE	Exp (*B*)	*p*
2019–2020			
Overwintering site			<0.001
Open-field	−0.269 ± 0.085	0.764	0.002
Semi-field	−0.180 ± 0.085	1.197	0.034
Establishment date			<0.001
30 October 2019	−1.097 ± 0.086	0.334	<0.001
19 November 2019	−1.041 ± 0.088	0.353	<0.001
Sex	−0.180 ± 0.067	0.835	0.008
2020–2021			
Overwintering site			<0.001
Open-field	0.917 ± 0.187	2.502	0.000
Semi-field	2.629 ± 0.214	13.862	0.000
Establishment date			<0.001
6 November 2020	0.008 ± 0.214	1.008	0.971
14 December 2020	−2.555 ± 0.230	0.078	0.000
Sex	−0.180 ± 0.067	0.835	0.008

**Table 5:** Results of the binary logistic regression testing the effects of overwintering site and establishment date on adult emergence rates of overwintering pupae. Pupae transferred into urban conditions on 5 December 2019 (season 2019/2020, top), respectively, on 30 December 2020 (season 2020/2021, below) from the baseline.

Source of Variance	*B* ± SE	Exp (*B*)	*p*
2019–2020			
Overwintering site			<0.001
Open-field	0.019 ± 0.113	1.019	0.865
Semi-field	0.367 ± 0.113	1.444	0.001
Establishment date			<0.001
30 October 2019	−1.552 ± 0.122	0.212	<0.001
19 November 2019	−2.958 ± 0.115	0.052	<0.001
2020–2021			
Overwintering site			<0.001
Open-field	0.669 ± 0.167	1.952	<0.001
Semi-field	1.980 ± 0.251	7.244	<0.001
Establishment date			<0.001
16 October 2020	−0.800 ± 0.205	0.449	<0.001
22 November 2020	−0.473 ± 0.213	0.623	0.026

**Table 6:** Explanatory variables of the Cox proportional hazard model on the adult emergence time that are related to the developmental period of overwintering pupae. *B* is the model coefficient and exp (*B*) shows the relative risk of adult survival at any given time. Pupae transferred to urban site on 18 December 2019 (season 2019/2020, top) and 30 December 2020 (season 2020/2021, below) from the baseline, respectively.

Source of Variance	*B* ± SE	Exp (*B*)	*p*
2019–2020			
Overwintering site			<0.001
Open-field	4.031 ± 0.361	56.292	<0.001
Semi-field	−2.991 ± 0.141	0.050	<0.001
Establishment date			<0.001
30 October 2019	5.545 ± 0.361	255.855	<0.001
19 November 2019	6.080 ± 0.360	437.211	<0.001
Over. Site * Establishment date			<0.001
Open-field * 30 October 2019	−3.687 ± 0.386	0.025	<0.001
Open-field * 19 November 2019	−3.148 ± 0.365	0.043	<0.001
Semi-field * 30 October 2019	0.572 ± 0.166	1.772	0.001
2020–2021			
Overwintering site			<0.001
Open-field	8.784 ± 40.919	0.000	0.830
Semi-field	−4.967 ± 0764	0.007	<0.001
Establishment date			<0.001
16 October 2020	6.391 ± 0.766	255.855	<0.001
22 November 2020	6.080 ± 0.360	437.211	<0.001
Over. Site * Establishment date			<0.001
Open-field * 16 October 2020	10.402 ± 40.920	32,931.043	0.799
Open-field * 22 November 2020	9.099 ± 40.920	8943.478	0.824

**Table 7:** Average longevity and lifetime fecundity of females emerged from overwintering pupae in the three overwintering sites at different establishment dates. Females remained at the same overwintering site as pupae from which they were obtained.

Overwintering Site	Establishment Date	N	Average ± SE	
			Lifespan (Days)	Fecundity (Eggs/Female)
Open-field	28 October 2019	30	104.1 ± 16.1	0.73 ± 0.4
	6 November 2019	37	143.6 ± 15.0	8.62 ± 3.6
	5 December 2019	22	104.3 ± 13.9	3.64 ± 0.5
Semi-field	28 October 2019	32	87.8 ± 8.8	0
	6 November 2019	37	41.5 ± 3.8	0
	5 December 2019	0		
Urban area	28 October 2019	35	168.2 ± 9.1	0.77 ± 0.4
	6 November 2019	39	137.9 ± 14.4	0.15 ± 0.1
	5 December 2019	30	110.8 ± 11.4	0.17 ± 0.2

## Data Availability

Data are contained within the article. The original contributions presented in this study are included in the article. Further inquiries can be directed to the corresponding authors.

## Supplementary Materials

The following supporting information can be downloaded at: https://www.mdpi.com/article/10.3390/insects16111104/s1, Figure S1: Age specific survival patterns of *Ceratitis capitata* males and females that were transferred in three overwintering sites on 30/10/2019, 19/11/2019, and 18/12/2019 during the winter season 2019–2020; Figure S2: Age specific survival patterns of *Ceratitis capitata* males and females that were transferred in three overwintering sites on 06/11/2020 and 14/12/2020 during the winter season 2020–2021.
