# Supplementary material for: Overwintering Capacity of the Mediterranean Fruit Fly in the Dalmatia Region of Croatia
Source: Insects. 2025 Oct 29;16(11):1104. doi: 10.3390/insects16111104 (PMC12653279; doi:10.3390/insects16111104)
Supplement: Supplementary file 1 [file insects-16-01104-s001.zip › insects-3762326-supplementary.pdf]

Supplementary Material

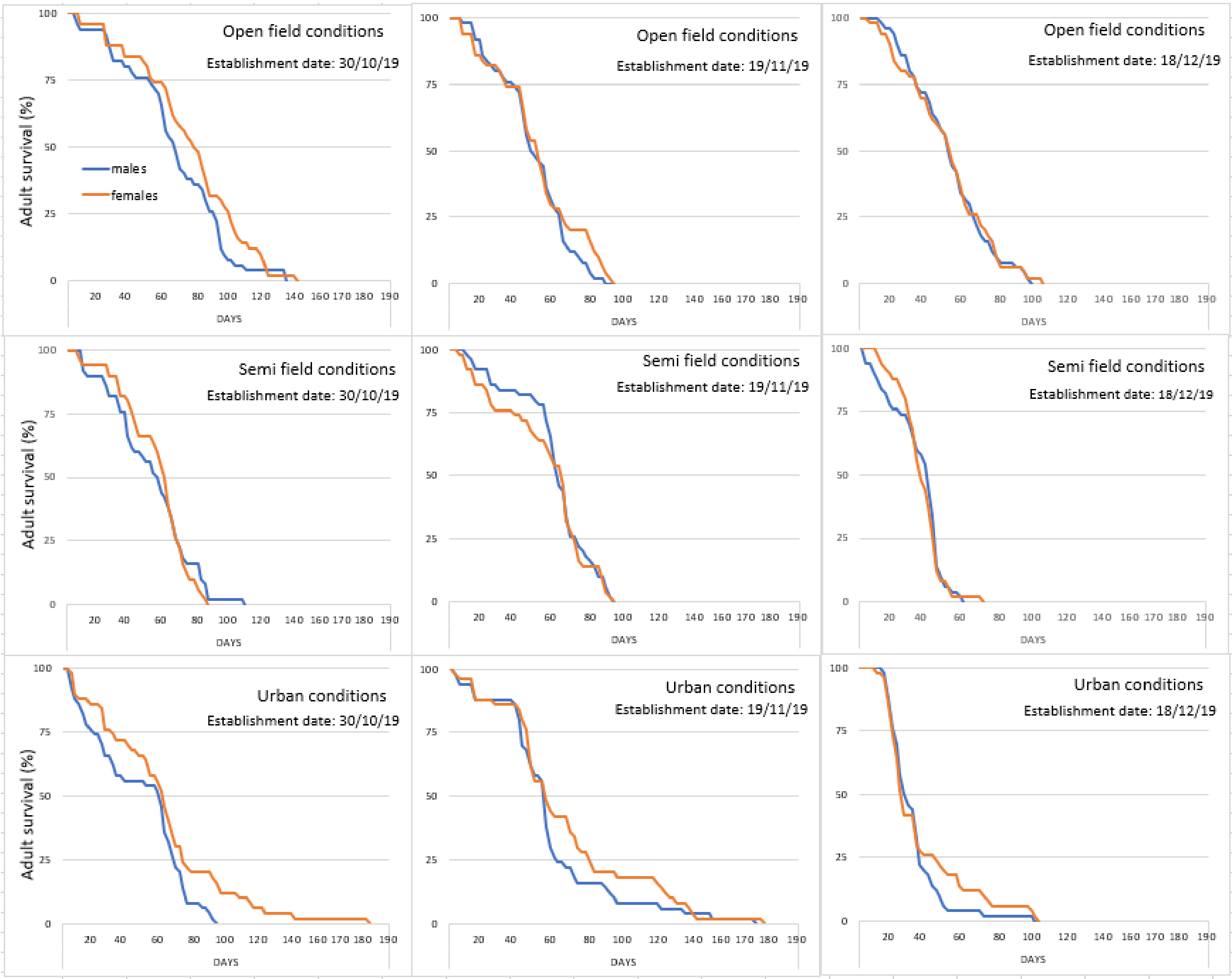

Figure S1. Age specific survival patterns of *Ceratitis capitata* males and females that were transferred in three overwintering sites on 30 October 2019, 19 November 2019, and 18 December 2019 during the winter season 2019–2020.

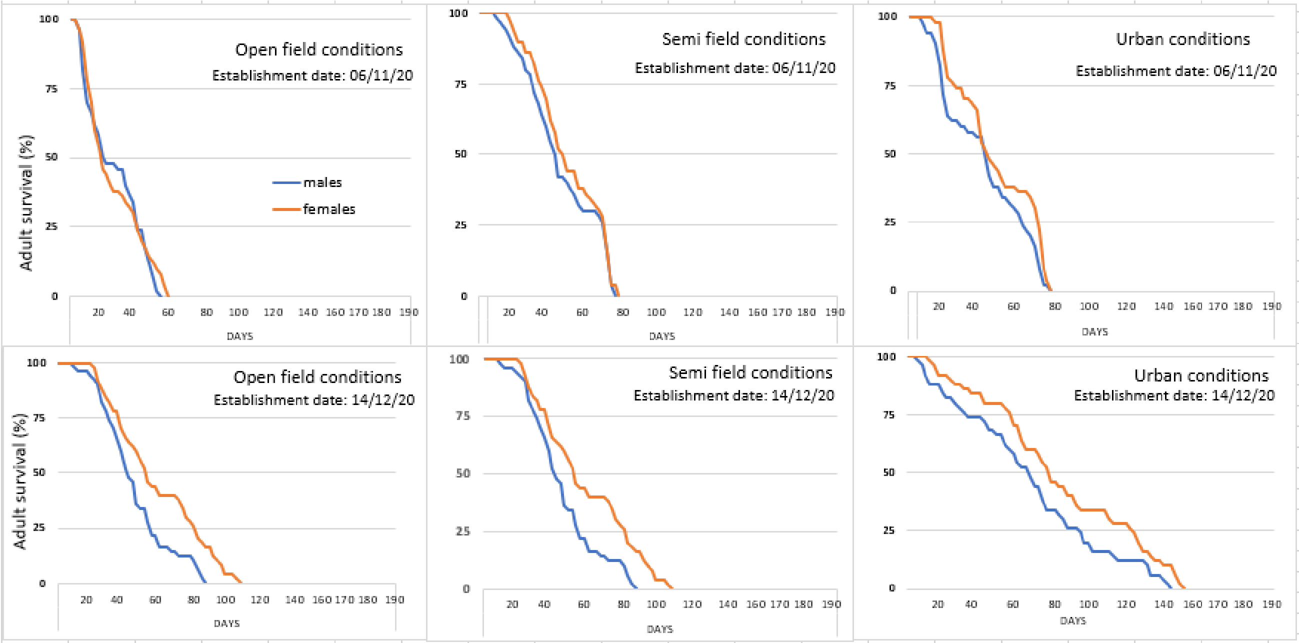

Figure S2. Age specific survival patterns of *Ceratitis capitata* males and females that were transferred in three overwintering sites on 6 November 2020 and 14 December 2020 during the winter season 2020–2021.
